# The future of MRI in radiation therapy: Challenges and opportunities for the MR community

**DOI:** 10.1002/mrm.29450

**Published:** 2022-09-21

**Authors:** Rosie J. Goodburn, Marielle E. P. Philippens, Thierry L. Lefebvre, Aly Khalifa, Tom Bruijnen, Joshua N. Freedman, David E. J. Waddington, Eyesha Younus, Eric Aliotta, Gabriele Meliadò, Teo Stanescu, Wajiha Bano, Ali Fatemi‐Ardekani, Andreas Wetscherek, Uwe Oelfke, Nico van den Berg, Ralph P. Mason, Petra J. van Houdt, James M. Balter, Oliver J. Gurney‐Champion

**Affiliations:** ^1^ Joint Department of Physics Institute of Cancer Research and Royal Marsden NHS Foundation Trust London United Kingdom; ^2^ Department of Radiotherapy University Medical Center Utrecht Utrecht Netherlands; ^3^ Department of Physics University of Cambridge Cambridge United Kingdom; ^4^ Cancer Research UK Cambridge Research Institute University of Cambridge Cambridge United Kingdom; ^5^ Department of Medical Biophysics University of Toronto Toronto Ontario Canada; ^6^ Elekta Limited Crawley United Kingdom; ^7^ Faculty of Medicine and Health, Sydney School of Health Sciences, ACRF Image X Institute The University of Sydney Sydney New South Wales Australia; ^8^ Department of Medical Physics, Odette Cancer Centre Sunnybrook Health Sciences Centre Toronto Ontario Canada; ^9^ Department of Medical Physics Memorial Sloan Kettering Cancer Center New York New York USA; ^10^ Unità Operativa Complessa di Fisica Sanitaria Azienda Ospedaliera Universitaria Integrata Verona Verona Italy; ^11^ Department of Radiation Oncology, University of Toronto and Medical Physics, Princess Margaret Cancer Centre University Health Network Toronto Ontario Canada; ^12^ Department of Physics Jackson State University (JSU) Jackson Mississippi USA; ^13^ SpinTecx Jackson Mississippi USA; ^14^ Department of Radiation Oncology Community Health Systems (CHS) Cancer Network Jackson Mississippi USA; ^15^ Department of Radiology University of Texas Southwestern Medical Center Dallas Texas USA; ^16^ Department of Radiation Oncology Netherlands Cancer Institute Amsterdam Netherlands; ^17^ Department of Radiation Oncology University of Michigan Ann Arbor Michigan USA; ^18^ Imaging and Biomarkers, Cancer Center Amsterdam, Amsterdam UMC University of Amsterdam Amsterdam Netherlands

**Keywords:** future, MR, radiation therapy, ISMRM workshop

## Abstract

Radiation therapy is a major component of cancer treatment pathways worldwide. The main aim of this treatment is to achieve tumor control through the delivery of ionizing radiation while preserving healthy tissues for minimal radiation toxicity. Because radiation therapy relies on accurate localization of the target and surrounding tissues, imaging plays a crucial role throughout the treatment chain. In the treatment planning phase, radiological images are essential for defining target volumes and organs‐at‐risk, as well as providing elemental composition (e.g., electron density) information for radiation dose calculations. At treatment, onboard imaging informs patient setup and could be used to guide radiation dose placement for sites affected by motion. Imaging is also an important tool for treatment response assessment and treatment plan adaptation. MRI, with its excellent soft tissue contrast and capacity to probe functional tissue properties, holds great untapped potential for transforming treatment paradigms in radiation therapy. The MR in Radiation Therapy ISMRM Study Group was established to provide a forum within the MR community to discuss the unmet needs and fuel opportunities for further advancement of MRI for radiation therapy applications. During the summer of 2021, the study group organized its first virtual workshop, attended by a diverse international group of clinicians, scientists, and clinical physicists, to explore our predictions for the future of MRI in radiation therapy for the next 25 years. This article reviews the main findings from the event and considers the opportunities and challenges of reaching our vision for the future in this expanding field.

## Abstract 2

Click here for author‐reader discussions

## 1 INTRODUCTION

Radiation therapy (RT), prescribed to ∼50% of cancer patients, is a major component of cancer treatment pathways.^1^, ^2^ The aim of RT is to deliver a sufficiently high dose of ionizing radiation to the tumor to control disease while limiting the dose to healthy tissues for minimal radiation toxicity. The most common RT modality, external‐beam RT, delivers megavoltage beams of X‐rays via a linear accelerator (Linac) mounted on a gantry that rotates around the patient. Carefully optimized treatment plans tailor beam profiles and photon intensities to focus the prescribed dose to the target volume(s) and minimize exposure to surrounding healthy tissue.^3^, ^4^ Typically, an RT course is run over 5 to 30 treatment sessions, called fractions that span 1 to 9 weeks. This article focuses on external‐beam RT, but some aspects are also applicable to other forms of RT, such as brachytherapy and proton therapy.

Imaging is performed at multiple points in the RT treatment chain with multiple objectives, which can be broadly categorized as: “delineation and dosing,” “guidance and targeting,” and “response and adaptation.”^5^ Delineation and dosing goals are met by acquiring CT simulation (CT‐Sim) images, often with complementary MRI or PET scans. Using these images, radiation oncologists delineate targets and radiosensitive normal tissues. Delineated targets and critical structures inform the treatment planning process that simulates and optimizes the planned dose distribution. Imaging is also used to inform guidance and targeting during treatment delivery. Onboard imaging is a vital component of modern Linac systems, which typically use mounted X‐ray systems capable of planar images and cone‐beam CT (CBCT) to align the tumor target with the position specified in the treatment plan through rigid couch adjustments each day of treatment.^6^ In some cases, imaging may also be used to adjust the radiation beams to compensate for internal anatomic changes,^7^ a process referred to as adaptive therapy. Because courses of RT span multiple fractions, there is room for tumor response assessment with CT, MRI, or PET to inform online or offline treatment plan adaptation.^8^, ^9^ These imaging techniques for response and adaptation objectives are active areas of clinical development.

**FIGURE 1:**
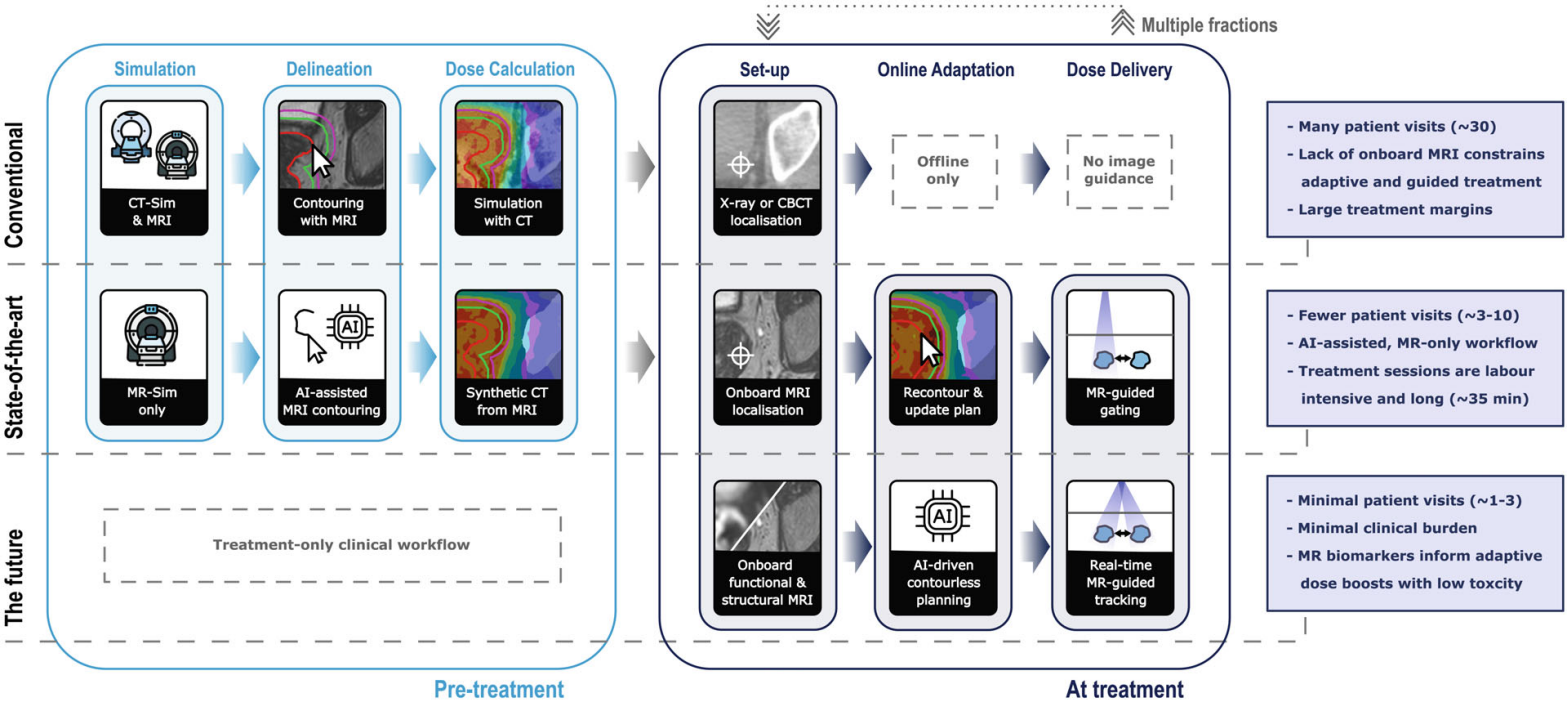
The role of imaging for radiation therapy (RT) in conventional, state‐of‐the‐art, and future workflows. The conventional workflow (top row) begins the pre‐treatment phase by scanning the patient in a computed tomography simulator (CT‐Sim), where the patient setup for treatment is simulated using the same flat‐top couch and positioning devices. MR scans are also acquired and registered to the CT images. Target volumes are delineated manually on MRI and dose distributions are simulated and optimized using the CT images. At treatment, patient setup on a Linac system is aided by onboard cone‐beam CT (CBCT) or planar X‐ray. The patient must return daily for repeated treatment fractions over the course of several weeks. The middle row illustrates a state‐of‐the‐art RT treatment chain. This MR‐only workflow replaces CT‐Sim with MR‐Sim, reducing the burden on hospitals and patients. Artificial intelligence (AI) assisted contouring increases the efficiency and reliability of delineation (2 DELINEATION). Treatment plans are calculated using synthetic CT generated from MR‐Sim images, eliminating CT‐MRI registration errors (3 DOSE CALCULATION). At treatment, hybrid MR‐Linac systems (6 HARDWARE) will facilitate the safe reduction of treatment margins via MRI‐informed adaptation to the daily anatomy and gated deliveries for moving targets (4 IMAGE GUIDANCE). Treatment sessions are more labor intensive than conventional treatments, but could lead to fewer patient visits overall (7 REDUCING PATIENT BURDEN). In the future (bottom row), an MR‐Linac‐only workflow without a pre‐treatment workup may be possible, where planning and treatment delivery is performed within minutes on the same system. Functional and structural MR imaging could inform AI‐driven algorithms to generate plans without input from clinicians. MR‐derived biomarkers (5 QUANTITATIVE MRI) hold the potential to establish new, contourless dose planning approaches, with information now available to inform the safe delivery of high‐dose boosts to targeted regions. Treatment plans could be delivered rapidly via real‐time MR‐guided tracking to continuously irradiate the target and safely (precisely) deliver dose distributions with steep spatial gradients. The presented workflow would greatly reduce patient and clinical burden (8 IMPLEMENTATION AND DISSEMINATION).

Although X‐ray and CT‐based technologies currently dominate imaging in RT, MRI is quickly growing in this application and has significant untapped potential to improve the field. Figure 1 illustrates conventional, state‐of‐the‐art, and our vision for the future of RT workflows. MRI's superior soft‐tissue visualization in comparison to CT will improve delineation and dosing, allowing for reduced uncertainty in target localization and, therefore, more accurate treatments with the ability to safely deliver higher doses.^10^, ^11^, ^12^, ^13^, ^14^ The availability of functional MRI, or quantitate MRI (qMRI), techniques, such as DWI and oxygen‐enhanced MRI (OE‐MRI) could add useful contrasts and may be able to identify high‐risk regions of the target that would benefit from dose boosts.^15^ New standards for guidance and targeting have been created by the recent availability of commercial MR‐Linac systems that allow concurrent imaging during treatment for MRI‐guided RT (MRgRT).^11^, ^16^, ^17^, ^18^, ^19^ Last, response and adaptation could be advanced through MRI's potential for quantifying tumor radiation sensitivity.^20^ Adapting dosing to treatment response between fractions may improve patient outcomes and could be achieved either using diagnostic MR systems^21^ or MR‐Linacs.^22^

This article is a summary of the findings from a 2021 workshop held by the ISMRM MR in RT study group on the future of MRI in RT. The aim of the workshop was to explore study group members' predictions for the future of this expanding field in 25 years' time. An international assembly of MRI and RT scientists, clinicians, and clinical physicists met virtually to discuss the long‐term opportunities and challenges of this expanding field of research. Given the nature of this topic, some predictions are based on current literature, but we also consider the consensus of expert opinions at the workshop, for which limited literature is available to cite. Here, we reach out to the MR community to elucidate the unmet needs that must be addressed for our vision in Figure 1 to become a reality.

It should be noted that because of the tremendous flexibility of machine learning approaches, these will often be at the core of this article's suggestions for overcoming the challenges of MRI in RT. However, where machine learning is applied in future clinical workflows, a consensus should be established on the validation, testing, and quality assurance (QA) of the techniques.^23^ Despite its great potential, machine learning should not be thought of as a silver bullet. It is well documented that machine learning solutions can behave poorly, for instance, when input data are out of distribution.^24^, ^25^ It is, therefore, important we are prepared to address this secondary set of challenges for clinical implementation.

## 2 DELINEATION

**FIGURE 2:**
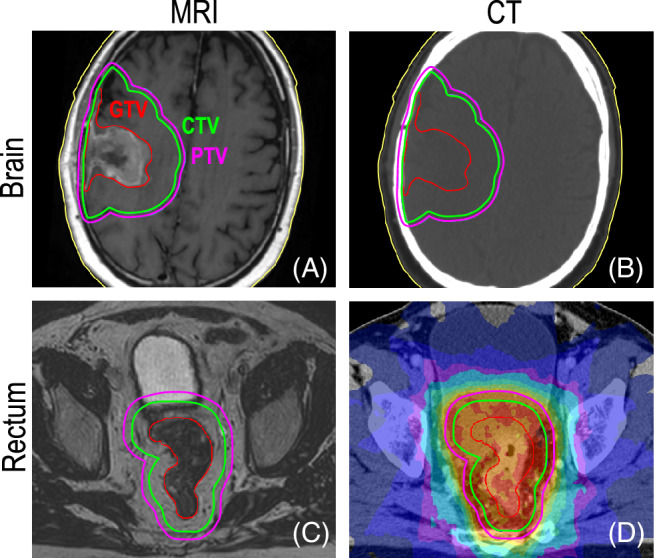
(A,B) Radiation treatment contours for a patient with brain cancer. Here, the gross tumor volume (GTV) (red) is contoured based on visible tumor tissue on MRI (A). The clinical target volume (CTV) (green) encompasses the GTV to account for subclinical spread not visible on imaging, based on anatomy and biological considerations. The planning target volume (PTV) (magenta) is designed to account for patient setup errors and beam inaccuracies, to ensure the prescribed dose is delivered to the CTV. The CT images (B) are not suitable for contouring here but are needed to provide elemental composition (e.g., eletron density) information for dose calculations. (C,D) Radiation treatment for a patient with rectal cancer. Again, the GTV is contoured based on MRI visibility (C). The MRI is registered to a CT image, which is used to calculate and optimize the planned dose distribution illustrated by the colorwash overlay (D). Note that the CTV‐PTV margin is large compared to the brain site treatment plan because of greater setup uncertainty and intrafraction motion.

Delineation, or contouring, is performed by radiation oncologists using a combination of CT‐Sim and co‐registered MRI or PET scans to outline a set of 3D contours (i.e., treatment targets and critical structures). These contours are defined by The International Commission on Radiation Units and Measurements^26^ guidelines, and include the gross tumor volume (GTV), clinical target volume (CTV), planning target volume (PTV), and selected normal tissues, termed the organs at risk (OARs).^27^ As illustrated in Figure 2, the GTV represents the extent of the primary tumor revealed by imaging and is outlined manually. The CTV extends the GTV to account for invisible, sub‐clinical spread. A predefined GTV‐to‐CTV margin is typically used, although, depending on the treatment site, the CTV may instead be manually contoured or defined by the entire involved tissue (e.g., the prostate). The purpose of the PTV is to ensure CTV coverage, and it is built by adding margins that account for uncertainties in delineation, patient setup, physiological motion, and treatment delivery.

### 2.1 Autocontouring

Despite being labor‐intensive^28^, ^29^ and frequently resulting in large inter‐ and intra‐observer variations,^30^, ^31^ delineation of target volumes and OAR structures is conventionally performed manually. Machine learning‐based, automated contouring (autocontouring) is one popular solution^32^, ^33^ that is currently being introduced in daily practice. It promises greater reproducibility and accuracy of delineated structures, with a substantial reduction in clinical burden. Today, the feasibility of autocontouring treatment targets^28^, ^34^, ^35^, ^36^, ^37^, ^38^, ^39^ and OARs^34^, ^40^, ^41^, ^42^ using MRI has been demonstrated and commercial solutions are rapidly being released. However, the success of autocontouring over the coming years will rest on balancing clinical, industrial, and regulatory interests.^43^

Automated contouring must be robust and flexible for clinical implementation to be feasible. For instance, autocontouring models should be able to rapidly adapt to new imaging protocols, without the need to obtain and annotate a new training set. Future solutions may include generating large sets of synthetic data with the desired contrast for training using generative adversarial networks^41^, ^44^, ^45^, ^46^ and/or use protocol‐agnostic networks.^47^, ^48^ Autocontouring tools should also be able to handle inter‐scanner differences, such as model, field strength, vendor, etc. Such flexibility would allow inter‐institutional cooperation and access to vast heterogeneous training data across multiple centers. Therefore, federated learning, a technique for training deep neural networks in a decentralized fashion without exchanging original data between sites, could provide an invaluable resource in developing autocontouring solutions.^49^

For MRgRT on the MR‐Linac, the need to achieve low‐latency autocontouring for mid‐treatment tumor tracking presents another set of challenges. Because adapting targets to real‐time changes make clinician approval infeasible, automated QA tools will be needed. Machine learning‐based techniques for detection of erroneous or anomalous delineations^50^ or delineation uncertainty maps offer solutions here.^51^, ^52^ State‐of‐the‐art, online planning assisted with these tools could augment human review in treatment planning and allow smaller treatment margins by reducing inter‐observer variation.

### 2.2 New contouring paradigms

An inherent limitation of RT delineation is that treatment volumes depend on the imaging modality or contrast used. Specifically, GTVs extend only as far as what can be revealed by the imaging and GTV‐to‐CTV margins must provide a conservative estimate of undetected microscopic expansion, sometimes several cm in magnitude.^53^ In the future, advanced application of cutting‐edge MRI methods (see 5 Quantitative MRI) may hold the key to safely reducing GTV‐CTV margins through improved visualization and/or understanding of the underlying biology. These advances might also allow novel contouring concepts to be implemented clinically, such as probabilistic margin optimisation^54^ or even contourless planning. With changes in delineation concepts (including autocontouring), new methods for performing clinical evaluation of contours will also be needed. Geometric measures for evaluating contours (e.g., Dice similarity index) will no longer be clinically relevant.^55^ Alternatively, dosimetric comparisons could be made compared with plans generated with ground truth reference contours.^55^, ^56^, ^57^, ^58^

The clinical implementation of new contouring paradigms relies on histopathological validation and large clinical trials so that standards and guidelines may be developed.^59^ In transitioning to new contouring paradigms, the MR and RT communities will need to first determine target definitions by asking what should be delineated and why. Second, we must establish whether a delineation task can reliably be achieved. Third, how new contours are used for dose prescription must be addressed. All three aspects would ideally form one consistent and robust clinical strategy.

### 2.3 Unmet needs

- Development and optimization of autocontouring methods that function robustly with heterogeneous inter‐institutional data;
- Clinical QA solutions for safe autocontouring for mid‐treatment tumor tracking; and
- A re‐examination of how target delineations are defined, in collaboration with the RT community.

## 3 DOSE CALCULATION

Dose calculation is the computation of energy deposited by ionizing radiation in the patient (i.e., radiation “dose”). Following delineation, treatment planning software is used to simulate the interaction between the patient and the planned treatment X‐ray beams. An iterative process is used to update the beams to optimize the calculated dose in tumor targets and OARs. To model photon scatter and absorption within the patient, information is needed on the tissue elemental composition (e.g., electron density), where this is conventionally derived from dedicated CT imaging. In state‐of‐the‐art RT and in the future, so‐called synthetic CT (sCT)—CT‐like images derived from MRI—will facilitate MRI‐only workflows^60^ and adaptive replanning on MR‐Linac systems^61^ by providing up‐to‐date sCT maps free from registration errors. In addition, hybrid PET/MR systems give rise to a similar need for attenuation correction,^62^ where the culmination of research in both areas advances sCT generation techniques.^63^

Although MRI cannot directly measure X‐ray attenuation, many techniques for generating sCT have been proposed in the literature^64^ and vendor‐provided solutions already exist for brain and pelvis.^65^, ^66^, ^67^ However, commercial sCT solutions are not available for more complex anatomies, such as the thorax, or tumor sites close to abnormal bony anatomy.^68^ Where most vendor solutions are based on bulk density overrides or atlases, recent sCT approaches in research use machine learning architectures, such as generative adversarial networks^63^, ^69^, ^70^, ^71^, ^72^ that may provide solutions for more challenging datasets and anatomic sites. As discussed in the previous section, successful clinical implementation of machine learning‐based methods for use in treatment planning will depend on their robustness to clinical variability, such that they meet the quality standards defined by consensus guidelines,^12^ and the development of suitable QA phantoms for end‐to‐end testing.

Today, sCT image volumes are designed to match the resolution and axial orientation of CT scans that are anticipated by treatment planning software. However, planning systems may soon be adapted to more conveniently handle other orientations that are facilitated by MRI.^73^ Later, 4D sCT or MRI‐based motion signals may inform the simulation software for more advanced treatment planning in moving anatomies.^74^

Looking further ahead, sCT may only be a stepping stone on the way to a new RT paradigm. Future pipelines may not directly reconstruct or display sCTs but, instead, feed *k*‐space data directly to the planning system algorithm to generate a treatment plan using predefined library matching or machine learning approaches. Conversely, reconstruction of sCT images might never leave treatment chains totally since intermediate representations could generate optimal performance.^75^ Furthermore, supervision of key intermediate steps is needed for QA purposes and so is likely to remain desirable for years to come.

### 3.1 Unmet needs

- 4D‐sCT methods suitable for adaptive MRgRT in complex anatomies, such as thoracic sites.

## 4 IMAGE GUIDANCE

Image guidance is the process of using imaging at the treatment phase to inform up‐to‐date localization of tumor targets and healthy tissues. Modern Linacs typically house onboard CBCT to facilitate alignment of the targets to the treatment plan model at the start of each fraction.^6^ However, conventional image guidance is limited by poor soft tissue contrast and lack of online motion characterization.^76^ Residual targeting errors are generally accounted for by the CTV‐to‐PTV margins, although large margins limit the dose that can be safely delivered while sparing nearby OARs.^77^ Hybrid MR‐Linac systems promise to allow reduced PTV sizes through the superior localization and targeting afforded by onboard MRI.^78^ Accurate, low‐latency motion characterization will facilitate gated treatment,^79^ tumor tracking during irradiation,^80^, ^81^, ^82^, ^83^, ^84^ and could enable real‐time adaptive replanning. Such precise treatments, delivered to smaller PTVs, will permit safe dose escalation^85^, ^86^ and hypofractionation^87^ to improve patient outcomes and clinical efficiency. Furthermore, management of bulk patient motion with real‐time MRI could remove the need for uncomfortable immobilization devices.

### 4.1 4D‐MRI

In the RT context today, 4D‐MRI generally refers to respiratory‐correlated 3D‐MRI, with image volumes acquired over several breathing cycles and retrospectively binned into respiratory phases.^88^ Potential applications of motion characterization using 4D‐MRI include onboard treatment plan adaptation and retrospective dose calculations, where 4D‐MRI serves as a precursor to volumetric real‐time imaging.^79^

In the future, respiratory‐correlated 4D‐MRI could be replaced by truly time‐resolved 4D‐MRI (i.e., volumetric real‐time imaging),^88^ with potential applications in tracking, gating, and real‐time dose monitoring. Current developments include using motion models built from prospectively acquired 4D‐MRI^89^ to rapidly generate synthetic 4D‐MRI updated by 2D imaging of the motion perpendicular to the treatment beam.^83^ Alternatively, the use of higher‐order surrogate signals^90^ can resolve signal characteristics beyond respiratory motion,^91^ enabling simultaneous resolution of peristaltic motion^92^ or cardiac motion to aid cardiac gating for MRgRT of ventricular tachycardia.^93^

### 4.2 Real‐time MRI

To fully realize the potential of motion management for MR‐guided adaptive targeting, low‐latency, high‐fidelity data for precise spatial–temporal localization is desirable. However, low‐latency goal differs for each motion type. For instance, cardiac motion is on the sub‐second scale, whereas organ filling extends over minutes.^76^ Recommendations have been made for MRI latencies of 200‐500 ms for respiratory motion,^94^ although how fast this could be and still make a clinical impact is an open question that must be revisited as research progresses. Currently, when mid treatment adaptation is desired, time‐resolved 2D‐MRI images are often obtained, rather than 4D‐MRI.

Real‐time adaptive image processing for MRI is an area of ongoing research.^95^ To minimize latency, the amount of acquired data per frame must be reduced. Potential solutions include the use of accurate spatiotemporal motion models,^96^, ^97^ suitable low‐rank subspace constraints,^98^ or sparsely sampled *k*‐space data interpreted by compressed sensing.^91^ However, for most of these accelerated MRI acquisition techniques, the gain in acquisition time results in longer reconstruction times. Fortunately, machine learning approaches that transfer computational processing to offline training of a neural network^99^, ^100^, ^101^, ^102^ may overcome long reconstruction times of accelerated acquisitions. In the future, latency for treatment planning and image guidance could be further reduced through use of patient representations composited from models that extract various representative states and their probabilistic variations. Tighter integration may gradually lift the need to exchange information between MRI scanners and Linacs in the form of images, opening opportunities for reducing latency through direct contour tracking from raw MRI data.^58^, ^103^

Opportunities and challenges of real‐time imaging for MRgRT are shared by interventional MRI.^104^ We should, therefore, ensure that these fields learn from one another as solutions are explored in the coming years. In addition, MR‐guided proton therapy will benefit from advances in MRgRT, where a full characterization of target and OAR motion is crucial because steep dose gradients exist not only perpendicular to the beam, but also along the direction of the beam.^105^

### 4.3 Unmet needs

- Low‐latency, high‐fidelity, precise spatial–temporal localization of target volume and organs at risk;
- Rapid online reconstruction of highly undersampled MRI data; and
- A tighter integration of MRI and RT systems for adaptive planning informed directly by *k*‐space data.

## 5 QUANTITATIVE MRI

Biomarkers derived from qMRI techniques allow for non‐invasive assessment of morphological, biological, and functional processes in tissue and so promise several key roles throughout RT workflows.^106^ First, qMRI could improve visualization for delineation by incorporating advanced contrast mechanisms. Second, qMRI biomarkers promise to provide metrics for RT response to allow adaptive treatment based on physiological responses^9^ (e.g., necrosis) that manifest earlier than anatomical imaging features.^21^, ^107^ For instance, changes in cell density—a well‐established marker for early response detection—can be measured indirectly using DWI for early response detection.^9^ Third, qMRI techniques may offer a surrogate for tissue dose sensitivity, such that treatment dose boosts can be informed and adapted according to baseline measurements.^108^ Several recent articles have been published on the use and level of evidence for different qMRI techniques in RT.^103^, ^106^, ^109^ We particularly refer to Table 1 in van Houdt et al.^103^

### 5.1 MRI‐derived biomarkers

An active area of MRI research that works to detect radiation sensitivity is the investigation of hypoxia, a well‐established and important prognostic marker for radioresistance. Hypoxic tissues require up to 3‐fold greater doses to achieve the same biological effectiveness.^110^ Although there are several MRI approaches for assessing oxygenation (pO_2_), they are predominately indirect. Tissue water T_1_ is sensitive to pO_2_ because the oxygen molecule is paramagnetic. The effect is small, but recent pre‐clinical work demonstrated the feasibility of stratifying tumors based on pre‐irradiation oxygen gas breathing to predict long term tumor control following radiation.^108^ Meanwhile, T_2_^*^ is strongly influenced by the concentration of deoxyhemoglobin. Perfusion is also an indirect marker for hypoxia, which can be measured using DCE, arterial spin labelling, or intravoxel incoherent motion. A more direct way of measuring pO_2_ is with dynamic oxygen‐17 MRI^111^; however, this technique is expensive and suffers from weak SNR, so has not been commonly investigated.

Other commonly investigated qMRI techniques for RT include CEST and MR spectroscopy.^103^ Recent analysis suggests radiation dose could be effectively adapted using a genomic‐adjusted radiation dose model^112^ and active investigations seek similar capabilities based on radiomics.^113^ Ultimately, we believe a combination of several techniques will allow complimentary information to be sampled on the state of the tumor. These data will allow clinicians to generate better personalized treatment plans than ever before, targeting dose to (hypoxic) radioresistant tumor regions and reducing dose to regions it is no longer needed.

We expect that the impact of qMRI development for RT will not only improve RT outcomes, but allow RT in cases that are currently considered unsuitable. For instance, lung cancer patients with severe lung function loss are often limited to surgery because of the risk of damage to remaining healthy tissue. However, with qMRI in combination with ventilation of hyperpolarized gases, functional regions of the lung can be clearly identified and considered, enabling RT as viable treatment in these patients.^114^, ^115^, ^116^, ^117^

### 5.2 From research tool to clinical tool

Currently, qMRI for RT is predominantly a research tool, with most work focusing on establishing a link between MRI and treatment response. To translate qMRI to clinical use, the next step will be establishing quantitative imaging biomarkers (QIBs) from qMRI parameters. The general imaging biomarker roadmap of O'Connor et al.^118^ provides a useful framework for these next steps, where there must be a transition from a promising QIB, to a potential QIB, and ultimately toward a clinically validated QIB.^119^

Today, evidence for QIBs in RT is limited. Complex logistics and the added patient burden of extra MRI examinations^103^ mean that analyses are often based on small patient cohorts or very few time points. To overcome these difficulties, functional imaging data for QIB studies could be collected on MRgRT systems at the time of treatment. Through systematic measurement of qMRI across treatment courses, large collaborative libraries could be built to detect which qMRI techniques generate truly prognostic QIBs. Such an initiative would require large, collaborative networks that include experts from both MRI and RT communities, such as the Elekta MR‐Linac consortium, to collect data prospectively and systematically over many years. To supplement this, robust data‐science frameworks should be established, which are often overlooked in qMRI studies.

When the prognostic value of a set of qMRI parameters has been systematically demonstrated in a large cohort, the next step of clinical validation is confirmation that the qMRI method also has predictive value (i.e., can be used to modify treatment). Investigations into predictive value can be conducted using interventional trials that adhere to the RT idea, development, exploration, assessment, and long‐term evaluation framework.^120^ Although we must first ensure that any unknowns are first solved, like how qMRI parameter maps are translated into the dose prescription.

To systematically study the relation between qMRI parameters, dose, and treatment response requires comparing qMRI with clinical outcome measures at different treatment dose levels. Some insight can be gained by comparing results between periods where guidelines for dose prescriptions changed or countries that prescribe differing treatment doses. Ultimately, however, qMRI validation requires randomized trials with variable dose. Setting up such trials is challenging because current dose levels are the accepted clinical standard. Changing doses could benefit some patients but could result in a worse outcome for others. Therefore, informed, careful patient selection, and close collaboration between qMRI experts and oncologists will be essential. In particular, radiation oncologists should have a more advanced understanding of the underlying qMRI mechanisms so that they can be comfortable in adapting treatment.

Initial efforts toward consensus guidelines for qMRI on MRgRT systems have recently begun.^22^ However, the current focus of qMRI in RT is on the target volume, where QIBs that monitor normal tissue toxicity^121^ could be further explored. To further develop guidelines for RT QIBs, there are opportunities to learn from and work with the diagnostic qMRI community, building on pre‐existing work. Such opportunities include initiatives for accurate and reproducible qMRI,^122^ learning from the Quantitative Imaging Network,^123^ and guidelines from the Quantitative Imaging Biomarker Alliance (QIBA) on DWI and DCE‐MRI.^124^ In adapting diagnostic recommendations for RT, we must remember that MR‐Linacs differ from conventional MRI systems.^125^, ^126^ For instance, images from MRgRT systems typically exhibit a lower SNR than those obtained using diagnostic devices and sometimes have unconventional field strengths.

### 5.3 Adaptive treatment

One major opportunity for qMRI that arose with the onset of MR‐Linac systems is daily tumor biology‐based treatments. For instance, the availability of real‐time qMRI techniques could improve RT efficacy by allowing treatment to be timed to when the tumor is at its most sensitive to irradiation, such as outside of hypoxic periods.^20^, ^127^, ^128^, ^129^, ^130^, ^131^, ^132^ MRI might also be used to directly enhance treatment. For instance, radiation sensitivity could be increased with drugs targeted to the tumor tissue with MRI, using a similar approach as MR targeting.^133^ Alternatively, hypoxia could be reduced by breathing hyperoxic or hyperbaric oxygen,^134^ with qMRI used to confirm normoxic status.

Another application of qMRI in RT could be real‐time visualization of biological dose. Because radiation dosimetry can be assessed in vitro using Bang gels, one can envisage extension to in vivo applications.^135^ Through a deeper understanding of the short‐term effects of dose on tissue, we may find an MRI contrast mechanism, such as CEST or DWI, is sensitive to the short‐term biological effect of the treatment beam. Such qMRI methods could be used for validation and adaptation of the planned treatment.

### 5.4 Protocol optimization

The image quality of qMRI is notoriously poor when compared to conventional MRI. Because multiple images must be obtained to model and measure signal changes, image resolutions are low despite long acquisition times. Therefore, clinicians often prefer conventional MR images for tumor assessments. Technical improvements in acquisition speed and image quality will greatly aid implementation of qMRI in clinical workflows.

For state‐of‐the‐art RT on MR‐Linacs, faster qMRI is imperative. Today, qMRI measurements are acquired during the opportunity‐time created by manual contouring. With the clinical adoption of autocontouring (see 2 Delineation), the time available for qMRI measurements for MRgRT will be shortened. Methods such as MR fingerprinting,^136^ model‐based image reconstruction,^137^ and MR‐spin tomography in time‐domain^138^ could enable substantially shorter acquisitions and could yield higher resolution qMRI images with improved accuracies. However, shorter acquisition times often come with a trade‐off of longer reconstruction times. For online applications, the solution may be machine learning‐based methods for rapid reconstruction^102^, ^139^, ^140^ and modeling.^141^, ^142^, ^143^ Ultimately, we may measure QIBs in tumors directly from undersampled raw *k*‐space data to meet the goal of real‐time monitoring and treatment adaptation.^144^

### 5.5 Unmet needs

- Established, standardized QIBs for RT derived from qMRI parameters;
- Demonstration of the predictive value of QIBs across large multi‐center cohorts;
- Accelerated pipelines for acquisition, reconstruction, and interpretation of qMRI; and
- Improved image quality of qMRI parameter maps.

## 6 HARDWARE

### 6.1 MRI for RT

Standalone MRI systems may be used for simulation imaging (i.e., MR‐Sim). Compared to conventional diagnostic MRI scanners, these pre‐treatment imaging systems must meet additional RT‐specific requirements.^145^ For instance, high spatial accuracy is important since geometrical image distortions can lead to under‐exposure of the tumor site and unnecessary dose to healthy structures.^144^ Geometric fidelity depends on magnetic field homogeneity and gradient linearity, which are typically worse at higher field strengths and can be compromised by the integration of the Linac system.^146^ Unlike for diagnostic MRI, the geometric fidelity of MR images is critically important in RT applications. The implementation of MRI for RT has, therefore, largely focused on minimizing and charactering^147^ distortions as new techniques and QA procedures were developed.^148^ Today, this issue is largely solved, but will remain an important factor to consider as the technology develops.

The installation of conventional MRI systems in RT departments can be complex and costly. Large scanner weights, the need to incorporate a quench pipe in shielding designs, and the undesirable interaction between MRI fringe fields and nearby Linacs are often challenging factors, and the need for MR‐Safe^149^ immobilization devices and other devices (e.g., intravenous‐contrast pumps) further adds to the cost. In addition, wide scanner bores are required to accommodate immobilization equipment.

Several recent developments can help adapt diagnostic systems for RT purposes. The industry has recently developed diagnostic MR scanners with low helium content (e.g., <8 L) that do not require a quench pipe, allowing for lower installation costs and a reduced environmental impact.^150^ Low‐field scanners are another example of these developments, which can improve geometric accuracy for RT,^151^ but this trade‐off must be considered with SNR and image quality losses below 1 T (particularly for qMRI). However, in the future, low‐field SNR may be significantly boosted through machine learning driven reconstruction.^152^

### 6.2 MR‐Linac systems

Hybrid MR‐Linac systems present a different set of engineering challenges. For instance, the influence of the MR system fringe field on the Linac must be minimized. In addition, the RF radiation originating from the Linac must be shielded from the MRI sub‐system, which must, in turn, be carefully design to meet radiation attenuation requirements.

Currently, two MR‐Linac solutions are commercially available, which have each taken different approaches to the integration of an MR scanner with RT beam‐generation components.^126^, ^153^ Both MR sub‐systems are based on diagnostic designs, which have been modified to meet RT workflow and dosimetric requirements while maintaining imaging performance (e.g., spatial integrity). The Unity (Elekta AB) MR device^154^ is based on a modified 1.5 T MRI (Philips Healthcare). The magnet is optimized to create a surrounding annulus of a low magnetic field to enable its decoupling from the rotating‐gantry‐mounted ferromagnetic components that include the beam generation sub‐systems. The magnet was also modified to create a radiation window by splitting the gradient coils.^155^ The MRIdian (ViewRay Inc) system houses a superconducting, 0.35‐T, split‐magnet design, using ferromagnetic shielding to isolate the Linac sub‐systems on the ring gantry from the magnet. In both vendor designs, the gap between the two magnet cryostat components permits megavoltage X‐ray beams to pass through with very little attenuation.^153^ Non‐commercially available MR‐Linac systems have focused on bi‐planar rotatable MR designs^156^ and the use of a standalone magnet with a non‐rotatable radiation beam.^157^

To date, ∼200 MR‐Linacs have been installed, which is limited compared to the global installed base of roughly 13 000 conventional Linacs. To provide improved access to MR‐Linacs, it is important that they become cheaper and simpler to use in the future. There are several challenges associated with the current designs. First, the MR magnet structure offers limited access to the patient table inside the bore. Second, the overall size, weight, and cost of the MR scanner adds complexity. The footprint of MR‐Linac systems may pose significant demands on the construction space required, greatly increasing installation costs. Third, only a very limited range of coils, with low numbers of coil elements, are available. Fourth, state‐of‐the‐art treatments, like volumetric‐modulated arc therapy, are not yet available for MR‐Linac systems.

As the MR‐Linac market grows, optimized components may start to differ from the mainstream diagnostic solutions to become more aligned with the unique needs of RT. Future iterations of MR‐Linac technology may include greater use of modeling for the MR magnet optimization problem, such that additional Linac structural and performance specifications are considered. MR‐Linac designs could also put more focus on requirements for maximizing patient access and minimizing hardware size.^158^ Vendors should facilitate easy MR‐Linac upgrades because these are essential to enabling rapid integration of novel treatment and imaging innovations.

Conversely, maintaining a similar blueprint could reduce overheads through the sharing of manufacturing, obsolescence, and supply costs. This could be aided further by focusing on open‐source hardware.^159^ Comparable designs could facilitate fast and easy translation of MRI solutions to the MR‐Linac domain. Another advantage is that when MR‐designs are similar, less retraining is required for in‐house radiology experts.

In addition to imaging performance, RF coil design for MRgRT applications must balance patient setup and dosimetric requirements.^160^ For instance, for some MR‐Linac designs, the RF coil elevates above the patient to reduce the excess surface dose at the expense of SNR.^161^ Future designs could instead reduce excess ssurface (skin) dose by constructing RF coils with inbuilt foam boluses, to allow the coils to be positioned closer to the patient for improved SNR.^162^ Low‐weight coils minimize body‐contour deformations and ease patient setup.^158^ Another important design consideration relates to the local beam attenuation and positioning of sensitive electronics, which limit the number of coil elements and, consequently, the parallel imaging capabilities of the MR subsystem. RF coils using high impedance capacitors could enable a high number of coil elements to be used while meeting the beam attenuation requirements.^163^, ^164^, ^165^ Alternatively, high‐density, disposable coils that allow electronics to be directly in the path of the radiation beam might be considered. Overall, many new coil designs could yet be exploited to optimize image quality for MRgRT, including wireless, flexible^165^, ^166^ and disposable RF coils, as well as inbuilt bolus designs.

### 6.3 Unmet needs

- Easier access to MR‐Sim and MR‐Linac systems: simpler installation, reduced costs, and reduced footprint; and
- Optimized MR‐Linac components that are more aligned with the unique needs of RT e.g., improved coil designs.

## 7 REDUCING PATIENT BURDEN

An important aspect for the success of MR in RT is minimizing patients' treatment burden. In addition to well‐being, patient burden^167^ includes the time, difficulty, and costs devoted to healthcare. Critical components of the treatment burden are the number of visits to the hospital (including travel time and costs), the duration and comfort of the treatment position.^168^

### 7.1 MR guided radiotherapy

The advent of MRgRT systems has initially increased treatment burden for patients since treatment session durations are substantially longer on MR‐Linacs than conventional Linacs. The average treatment time for prostate cancer has increased from 15 to 20 min on CBCT‐Linacs^169^ to 45 min on MR‐Linacs.^170^ Liver treatment on the MR‐Linac is particularly long, ranging from 60 to 90 min.^171^ Although 45 min is generally acceptable on diagnostic MRI scanners, MRgRT patients are set up in the treatment position, which can include a hard, flat tabletop, fixation devices (e.g., closely fitted full‐head masks), and uncomfortable positions (e.g., arms above the head).^73^ Furthermore, patients treated on MR‐Linacs experience increased MRI‐related acoustic noise^172^ and anxiety because of limited space.^173^, ^174^ With the many repeated MRgRT sessions throughout an RT course, acoustic noise has a more substantial impact on hearing than for a one‐off diagnostic MRI examination.

In the future, MRgRT on MR‐Linac systems presents several opportunities to reduce patient discomfort. First, adaptive planning using onboard MR imaging could allow for more comfortable treatment couches, where a hard, flat tabletop is no longer needed for consistent setup. Second, future MR‐Linac models could be developed with wider bores to reduce claustrophobia and aid access to the patient. Third, uncomfortable setup devices may be rendered unnecessary with online MRI tracking.^101^ Fourth, future developments in MRI, tumor tracking, and gated deliveries could remove the need for breath‐hold imaging and treatment deliveries.^175^

### 7.2 Hypofractionation

Hypofractionation, increasing the dose per fraction and reducing the number of fractions, allows a biologically similar treatment plan to be delivered in fewer hospital visits.^171^ However, a major challenge of hypofractionated approaches is that treatment becomes more sensitive to patient setup errors. MR‐Linac systems promise to make setup errors smaller and overcome these limitations. Consequently, clinicians are currently attempting to increase the dose per fraction in several MRgRT protocols.^176^, ^177^ By further improving image quality at planning and real‐time monitoring during treatment, we could further reduce uncertainties and gain confidence in continuing the reduction of fraction numbers. However, it should be noted that spreading treatment over multiple fractions can allow for the repair of healthy tissues between sessions, reducing potential toxicity. Furthermore, while hypofractionated treatment regimens have shown dramatic improvements to treatment response for many disease sites,^178^, ^179^, ^180^ these results are ionly possible with excellent geometric precision and, in some instances, tumor dose spread must be more heterogeneous to ensure normal issues are preserved.

Next‐generation RT workflows could be “one‐stop‐shop”, only requiring 1 to 3 patient hospital vistis. In these workflows, once the patient is set up on the MR‐Linac, fast MRI scans are acquired, target volumes and OARs are automatically contoured and, within seconds, an automated, single‐fraction, high‐dose treatment plan is developed and delivered, all during a single visit (Figure 1). Such an approach would increase clinical throughput and improve patient experience, especially for palliative patients. However, at present, “day‐one” treatment planning would require substantial clinical resources and automation is strongly desired.

### 7.3 Unmet needs

- Increased patient comfort via removal of uncomfortable elements from MRgRT treatment chains, such as hard tabletops, immobilization devices, and breath‐holding;
- Faster imaging and treatment to reduce time in the machine; and
- Improved image quality at planning and real‐time monitoring during treatment to improve confidence in hypofractionation.

## 8 IMPLEMENTATION AND DISSEMINATION

The implementation of MRI in RT will be accelerated and steered by the introduction of MRgRT on MR‐Linacs. Currently, however, only a few RT centers have MRI scanners installed in the RT department and MR‐Linacs only account for a small fraction (∼1.5%) of all treatment machines in clinical use. We expect that developments will initially take different directions for non‐academic and academic centers. In non‐academic centers with MR‐Sim only, the focus will be on targeting accuracy. For non‐academic centers with an MR‐Linac, this will be combined with fast and automated hypofractionated RT and target tracking, where hypofractionation greatly reduces the cost of RT. In academic centers, experiments will focus heavily on development for qMRI methods. As the use of MRI for RT increases, guidelines^145^ for its practical implementation should be reviewed and updated.

### 8.1 Clinical burden

Operating an MR‐Linac currently requires a large team of clinical and technical experts. Centers with MR‐Linacs often require an on‐site clinician for recontouring, two dual‐trained RT‐MR technologists to drive treatment, an on‐call MR‐RT physicist, and a large group of physicists available for quality control and maintenance. Even where the additional facilities and expertise required were minimal, the increased strain on staff resources caused by using an MR‐Linac is often significant, with treatment times typically doubling those of conventional systems.^109^ The cost of developing and maintaining new support teams for MR‐Linac treatments is manageable for large cancer therapy centers, but could be prohibitive for smaller (2‐3 Linacs), community‐based radiation therapy centers, which are typical across Europe and the United States.^181^, ^182^ We, therefore, predict that over the next few years, MRgRT will predominantly be conducted at larger specialized centers.

For the dissemination of MRgRT and MR‐Sim to non‐academic centers and for long‐term usage in academic centers to be successful, logistic, environmental, and staff burdens must be reduced. Efforts should be made to reduce treatment times and simplify the operation of the MR‐Linac. Simple solutions to reducing clinical burden include transferring staff training to external parties and investing in AI‐assisted workflows. Standardized MRI acquisitions for treatment planning and motion monitoring combined with AI‐driven MRI‐scanning methods reduce the complexity of MR knowledge needed by radiographers.^183^ Similarly, AI‐driven contouring and treatment planning will greatly reduce the time and staff requirements for on‐table plan adaptation of treatments.^184^ Looking further ahead, the operational burden on the physics staff could be reduced by increased automation of patient treatment and QA procedures and highly hypofractionated treatment courses.^176^, ^185^ Such a workflow would resemble the ultimate minimization in operational burden and drive the uptake of MRgRT across all radiation oncology centers. It is of vital importance that clinicians are included in the introduction of these new approaches since automated workflows remove clinical decision making and hypofractionated treatments deviate from the current clinical standard.

### 8.2 Unmet needs

- Standardization of MRI acquisitions for treatment planning and motion monitoring;
- Collaborative development of automated workflows by researchers and clinical teams; and
- Easy (or automated) operation of MRI and MRgRT systems.

## 9 CONCLUDING REMARKS

MRI has become indispensable in modern RT pipelines and its role is expected to grow. Advances in tumor delineation, onboard image guidance, and imaging biomarkers afforded by MRI promise to transform RT over the next 25 years. With this paradigm shift, a rich spectrum of new challenges and opportunities is presented for the MR community.

## FUNDING INFORMATION

KWF Kankerbestrijding (Dutch Cancer Society), Grant/Award Number: KWF‐UVA 2021‐13785; National Institutes of Health, Grant/Award Number: P30 CA142543; Cancer Institute NSW, Grant/Award Number: ECF/1015. Cancer Research UK program, Grant/Award Number: C33589/A28284

## References

[1] Lievens Y , Borras JM , Grau C . Provision and use of radiotherapy in Europe. Mol Oncol. 2020;14:1461‐1469. 32293084 10.1002/1878-0261.12690 PMC7332207

[2] Zubizarreta E , van Dyk J , Lievens Y . Analysis of global radiotherapy needs and costs by geographic region and income level. Clin Oncol. 2017;29:84‐92. 10.1016/j.clon.2016.11.011 27939337

[3] Teoh M , Clark CH , Wood K , Whitaker S , Nisbet A . Volumetric modulated arc therapy: a review of current literature and clinical use in practice. Br J Radiol. 2011;84:967‐996. 22011829 10.1259/bjr/22373346 PMC3473700

[4] Burman C , Chui C‐S , Kutcher G , et al. Planning, delivery, and quality assurance of intensity‐modulated radiotherapy using dynamic multileaf collimator: a strategy for large‐scale implementation for the treatment of carcinoma of the prostate. Int J Radiat Oncol Biol Phys. 1997;39:863‐873. 9369136 10.1016/s0360-3016(97)00458-6

[5] Dawson LA , Ménard C . Imaging in radiation oncology: a perspective. Oncologist. 2010;15:338‐349. 20413639 10.1634/theoncologist.2009-S106 PMC3227970

[6] Cho PS , Johnson RH , Griffin TW . Cone‐beam CT for radiotherapy applications. Phys Med Biol. 1995;40:1863‐1883. 8587937 10.1088/0031-9155/40/11/007

[7] Nijkamp J , Pos FJ , Nuver TT , et al. Adaptive radiotherapy for prostate cancer using kilovoltage cone‐beam computed tomography: first clinical results. Int J Radiat Oncol Biol Phys. 2008;70:75‐82. 17869445 10.1016/j.ijrobp.2007.05.046

[8] Mali SB . Adaptive radiotherapy for head neck cancer. J Maxillofac Oral Surg. 2016;15:549‐554. 27833352 10.1007/s12663-016-0881-y PMC5083691

[9] Morgan HE , Sher DJ . Adaptive radiotherapy for head and neck cancer. Cancers Head Neck. 2020;5:1‐16. 31938572 10.1186/s41199-019-0046-z PMC6953291

[10] Gurney‐Champion OJ , Versteijne E , van der Horst A , et al. Addition of MRI for CT‐based pancreatic tumor delineation: a feasibility study. Acta Oncol. 2017;56:923‐930. 28375667 10.1080/0284186X.2017.1304654

[11] Grégoire V , Guckenberger M , Haustermans K , et al. Image guidance in radiation therapy for better cure of cancer. Mol Oncol. 2020;14:1470‐1491. 32536001 10.1002/1878-0261.12751 PMC7332209

[12] Glide‐Hurst CK , Paulson ES , McGee K , et al. Task group 284 report: magnetic resonance imaging simulation in radiotherapy: considerations for clinical implementation, optimization, and quality assurance. Med Phys. 2021;48:e636‐e670. 33386620 10.1002/mp.14695 PMC8761371

[13] Rasch C , Barillot I , Remeijer P , Touw A , van Herk M , Lebesque J . Definition of the prostate in CT and MRI: a multi‐observer study. Int J Radiat Oncol Biol Phys. 1999;43:57‐66. 9989514 10.1016/s0360-3016(98)00351-4

[14] Rasch C , Keus R , Pameijer FA , et al. The potential impact of CT‐MRI matching on tumor volume delineation in advanced head and neck cancer. Int J Radiat Oncol Biol Phys. 1997;39:841‐848. 9369132 10.1016/s0360-3016(97)00465-3

[15] van der Heide UA , Houweling AC , Groenendaal G , Beets‐Tan RGH , Lambin P . Functional MRI for radiotherapy dose painting. Magn Reson Imaging. 2012;30:1216‐1223. 22770686 10.1016/j.mri.2012.04.010 PMC5134673

[16] Lagendijk JJW , Raaymakers BW , Raaijmakers AJE , et al. MRI/linac integration. Radiother Oncol. 2008;86:25‐29. 18023488 10.1016/j.radonc.2007.10.034

[17] Chandarana H , Wang H , Tijssen R , Das IJ . Emerging role of MRI in radiation therapy. J Magn Reson Imaging. 2018;48:1468‐1478. 30194794 10.1002/jmri.26271 PMC6986460

[18] Koste JR , van S d , Palacios MA , et al. MR‐guided gated stereotactic radiation therapy delivery for lung, adrenal, and pancreatic tumors: a geometric analysis. Int J Radiat Oncol Biol Phys. 2018;102:858‐866. 30061007 10.1016/j.ijrobp.2018.05.048

[19] Ugurluer G , Mustafayev TZ , Gungor G , et al. Stereotactic MR‐guided online adaptive radiation therapy (SMART) for the treatment of liver metastases in oligometastatic patients: initial clinical experience. Radiat Oncol J. 2021;39:33‐40. 33794572 10.3857/roj.2020.00976 PMC8024184

[20] O'Connor JPB , Robinson SP , Waterton JC . Imaging tumour hypoxia with oxygen‐enhanced MRI and BOLD MRI. Br J Radiol. 2019;92:20180642. 30272998 10.1259/bjr.20180642 PMC6540855

[21] Wong KH , Panek R , Dunlop A , et al. Changes in multimodality functional imaging parameters early during chemoradiation predict treatment response in patients with locally advanced head and neck cancer. Eur J Nucl Med Mol Imaging. 2018;45:759‐767. 29164301 10.1007/s00259-017-3890-2 PMC5978912

[22] Kooreman ES , van Houdt PJ , Nowee ME , et al. Feasibility and accuracy of quantitative imaging on a 1.5 T MR‐linear accelerator. Radiother Oncol. 2019;133:156‐162. 30935572 10.1016/j.radonc.2019.01.011

[23] Huynh E , Hosny A , Guthier C , et al. Artificial intelligence in radiation oncology. Nat Rev Clin Oncol. 2020;17:771‐781. 32843739 10.1038/s41571-020-0417-8

[24] Cohen JP , Cao T , Viviano JD , et al. Problems in the deployment of machine‐learned models in health care. Can Med Assoc J. 2021;193:E1391‐E1394. 34462316 10.1503/cmaj.202066 PMC8443295

[25] Oakden‐Rayner L , Dunnmon J , Carneiro G , Re C . Hidden stratification causes clinically meaningful failures in machine learning for medical imaging. Proceedings of the ACM Conference on Health, Inference, and Learning. ACM; 2020:151‐159. 10.1145/3368555.3384468 PMC7665161 33196064

[26] Stroom JC , Heijmen BJM . Geometrical uncertainties, radiotherapy planning margins, and the ICRU‐62 report. Radiother Oncol. 2002;64:75‐83. 12208578 10.1016/s0167-8140(02)00140-8

[27] Burnet NG , Thomas SJ , Burton KE , Jefferies SJ . Defining the tumour and target volumes for radiotherapy. Cancer Imaging. 2004;4:153‐161. 18250025 10.1102/1470-7330.2004.0054 PMC1434601

[28] Lin L , Dou Q , Jin YM , et al. Deep learning for automated contouring of primary tumor volumes by MRI for nasopharyngeal carcinoma. Radiology. 2019;291:677‐686. 30912722 10.1148/radiol.2019182012

[29] McCarroll RE , Beadle BM , Balter PA , et al. Retrospective validation and clinical implementation of automated contouring of organs at risk in the head and neck: a step toward automated radiation treatment planning for low‐ and middle‐income countries. J Global Oncol. 2018;4:1‐11. 10.1200/JGO.18.00055 PMC6223488 30110221

[30] Vinod SK , Jameson MG , Min M , Holloway LC . Uncertainties in volume delineation in radiation oncology: a systematic review and recommendations for future studies. Radiother Oncol. 2016;121:169‐179. 27729166 10.1016/j.radonc.2016.09.009

[31] Huq MS , Fraass BA , Dunscombe PB , et al. The report of task group 100 of the AAPM: application of risk analysis methods to radiation therapy quality management. Med Phys. 2016;43:4209‐4262. 27370140 10.1118/1.4947547 PMC4985013

[32] Sharp G , Fritscher KD , Pekar V , et al. Vision 20/20: perspectives on automated image segmentation for radiotherapy. Med Phys. 2014;41:05092. 10.1118/1.4871620 PMC4000389 24784366

[33] Oktay O , Nanavati J , Schwaighofer A , et al. Evaluation of deep learning to augment image‐guided radiotherapy for head and neck and prostate cancers. JAMA Netw Open. 2020;3:e2027426. 33252691 10.1001/jamanetworkopen.2020.27426 PMC7705593

[34] Cha E , Elguindi S , Onochie I , et al. Clinical implementation of deep learning contour autosegmentation for prostate radiotherapy. Radiother Oncol. 2021;159:1‐7. 33667591 10.1016/j.radonc.2021.02.040 PMC9444280

[35] Lin M , Momin S , Lei Y , et al. Fully automated segmentation of brain tumor from multiparametric MRI using 3D context deep supervised U‐net. Med Phys. 2021;48:4365‐4374. 34101845 10.1002/mp.15032 PMC11752426

[36] Rodríguez Outeiral R , Bos P , Al‐Mamgani A , Jasperse B , Simões R , van der Heide UA . Oropharyngeal primary tumor segmentation for radiotherapy planning on magnetic resonance imaging using deep learning. Phys Imaging Radiat Oncol. 2021;19:39‐44. 34307917 10.1016/j.phro.2021.06.005 PMC8295848

[37] Li J , Chen H , Li Y , Peng Y . A novel network based on densely connected fully convolutional networks for segmentation of lung tumors on multi‐modal MR images. Proceedings of the 2019 International Conference on Artificial Intelligence and Advanced Manufacturing ‐ AIAM 2019. ACM Press; 2019:1‐5.

[38] Trebeschi S , van Griethuysen JJM , Lambregts DMJ , et al. Deep learning for fully‐automated localization and segmentation of rectal cancer on multiparametric MR. Sci Rep. 2017;7:1‐9. 28706185 10.1038/s41598-017-05728-9 PMC5509680

[39] Lustberg T , van Soest J , Gooding M , et al. Clinical evaluation of atlas and deep learning based automatic contouring for lung cancer. Radiother Oncol. 2018;126:312‐317. 29208513 10.1016/j.radonc.2017.11.012

[40] Liu Z , Liu X , Xiao B , et al. Segmentation of organs‐at‐risk in cervical cancer CT images with a convolutional neural network. Phys Med. 2020;69:184‐191. 31918371 10.1016/j.ejmp.2019.12.008

[41] Kieselmann JP , Fuller CD , Gurney‐Champion OJ , Oelfke U . Cross‐modality deep learning: contouring of MRI data from annotated CT data only. Med Phys. 2021;48:1673‐1684. 33251619 10.1002/mp.14619 PMC8058228

[42] Gurney‐Champion OJ , Kieselmann J , Wong K , Harrington K , Oelfke U . Rapid and accurate automatic contouring of quantitative diffusion‐weighted MRI using a deep convolutional neural network. In: 7th MR in RT Symposium 2019.

[43] Mir R , Kelly SM , Xiao Y , et al. Organ at risk delineation for radiation therapy clinical trials: global harmonization group consensus guidelines: GHG OAR consensus contouring guidance. Radiother Oncol. 2020;150:30‐39. 32504762 10.1016/j.radonc.2020.05.038

[44] Jiang J , Hu YC , Tyagi N , et al. Tumor‐aware, adversarial domain adaptation from CT to MRI for lung cancer segmentation. Lecture Notes in Computer Science (including subseries Lecture Notes in Artificial Intelligence and Lecture Notes in Bioinformatics) 2018;11071 LNCS:777–785. 10.1007/978-3-030-00934-2_86 PMC6169798 30294726

[45] Cai J , Zhang Z , Cui L , Zheng Y , Yang L . Towards cross‐modal organ translation and segmentation: a cycle‐ and shape‐consistent generative adversarial network. Med Image Anal. 2019;52:174‐184. 30594770 10.1016/j.media.2018.12.002

[46] Yu B , Zhou L , Wang L , Fripp J , Bourgeat P . 3D cGAN based cross‐modality MR image synthesis for brain tumor segmentation. In: 2018 IEEE 15th International Symposium on Biomedical Imaging (ISBI 2018). IEEE; 2018: 626–630.

[47] Meyer MI , de la Rosa E , Barros N , Paolella R , van Leemput K , Sima DM . An augmentation strategy to mimic multi‐scanner variability in MRI. In: 2021 IEEE 18th International Symposium on Biomedical Imaging (ISBI). IEEE; 2021. pp. 1196–1200.

[48] Billot B , Greve D , van Leemput K , Fischl B , Iglesias JE , Dalca A A Learning Strategy for Contrast‐agnostic MRI Segmentation. arXiv preprint arXiv:2003.01995 2020.

[49] Rieke N , Hancox J , Li W , et al. The future of digital health with federated learning. NPJ Digit Med. 2020;3:1‐7. 33015372 10.1038/s41746-020-00323-1 PMC7490367

[50] Nourzadeh H , Hui C , Ahmad M , et al. Knowledge‐based quality control of organ delineations in radiation therapy. Med Phys. 2022;49:1368‐1381. 35028948 10.1002/mp.15458 PMC13648192

[51] Zhao G , Liu F , Oler JA , Meyerand ME , Kalin NH , Birn RM . Bayesian convolutional neural network based MRI brain extraction on nonhuman primates. Neuroimage. 2018;175:32‐44. 29604454 10.1016/j.neuroimage.2018.03.065 PMC6095475

[52] Kendall A , Badrinarayanan V , Cipolla R . Bayesian segnet: Model uncertainty in deep convolutional encoder‐decoder architectures for scene understanding. In: British Machine Vision Conference 2017, BMVC 2017. BMVA Press; 2017.

[53] Kruser TJ , Bosch WR , Badiyan SN , et al. NRG brain tumor specialists consensus guidelines for glioblastoma contouring. J Neurooncol. 2019;143:157‐166. 30888558 10.1007/s11060-019-03152-9 PMC6483830

[54] Tilly D , Holm Å , Grusell E , Ahnesjö A . Probabilistic optimization of dose coverage in radiotherapy. Phys Imaging Radiat Oncol. 2019;10:1‐6. 33458260 10.1016/j.phro.2019.03.005 PMC7807558

[55] Voet PWJ , Dirkx MLP , Teguh DN , Hoogeman MS , Levendag PC , Heijmen BJM . Does atlas‐based autosegmentation of neck levels require subsequent manual contour editing to avoid risk of severe target underdosage? A dosimetric analysis. Radiother Oncol. 2011;98:373‐377. 21269714 10.1016/j.radonc.2010.11.017

[56] van Rooij W , Dahele M , Ribeiro Brandao H , Delaney AR , Slotman BJ , Verbakel WF . Deep learning‐based delineation of head and neck organs at risk: geometric and dosimetric evaluation. Int J Radiat Oncol Biol Phys. 2019;104:677‐684. 30836167 10.1016/j.ijrobp.2019.02.040

[57] Kieselmann JP , Kamerling CP , Burgos N , et al. Geometric and dosimetric evaluations of atlas‐based segmentation methods of MR images in the head and neck region. Phys Med Biol. 2018;63:145007. 29882749 10.1088/1361-6560/aacb65 PMC6296440

[58] Eldesoky AR , Francolini G , Thomsen MS , et al. Dosimetric assessment of an atlas based automated segmentation for loco‐regional radiation therapy of early breast cancer in the Skagen trial 1: a multi‐institutional study. Clin Transl Radiat Oncol. 2017;2:36. 29657998 10.1016/j.ctro.2017.01.004 PMC5893527

[59] Ligtenberg H , Jager EA , Caldas‐Magalhaes J , et al. Modality‐specific target definition for laryngeal and hypopharyngeal cancer on FDG‐PET, CT and MRI. Radiother Oncol. 2017;123:63‐70. 28259450 10.1016/j.radonc.2017.02.005

[60] Kerkmeijer LGW , Maspero M , Meijer GJ , van der Voort van Zyp JRN , de Boer HCJ , van den Berg CAT . Magnetic resonance imaging only workflow for radiotherapy simulation and planning in prostate cancer. Clin Oncol. 2018;30:692‐701. 10.1016/j.clon.2018.08.009 30244830

[61] Persson E , Emin S , Scherman J , et al. Investigation of the clinical inter‐observer bias in prostate fiducial marker image registration between CT and MR images. Radiat Oncol. 2021;16:150. 34399806 10.1186/s13014-021-01865-8 PMC8365967

[62] Chen Y , An H . Attenuation correction of PET/MR imaging. Magn Reson Imaging Clin N Am. 2017;25:245‐255. 28390526 10.1016/j.mric.2016.12.001 PMC5385843

[63] Spadea MF , Maspero M , Zaffino P , Seco J . Deep learning based synthetic‐CT generation in radiotherapy and PET: a review. Med Phys. 2021;48:6537‐6566. 34407209 10.1002/mp.15150

[64] Johnstone E , Wyatt JJ , Henry AM , et al. Systematic review of synthetic computed tomography generation methodologies for use in magnetic resonance imaging–only radiation therapy. Int J Radiat Oncol Biol Phys. 2018;100:199‐217. 29254773 10.1016/j.ijrobp.2017.08.043

[65] Tyagi N , Fontenla S , Zelefsky M , et al. Clinical workflow for MR‐only simulation and planning in prostate. Radiat Oncol. 2017;12:1‐12. 28716090 10.1186/s13014-017-0854-4 PMC5513123

[66] Philips . Unleash the real power of MR simulation: MRCAT Brain. https://www.documents.philips.com/assets/20200706/90a01c146c864ad183c1abf000c82c3d.pdf?_gl=1*1093euc*_ga*MzIwNzM5Nzg3LjE2Mjg1NTAxODc.*_ga_2NMXNNS6LE*MTYyODg4NjE4My4yLjAuMTYyODg4NjIxNS4yOA.&_ga=2.195699172.99280882.1628886184‐320739787.1628550187. Published 2020. Accessed August 25, 2021.

[67] Siemens . MR‐only RT planning for the brain and pelvis with synthetic CT (white paper). https://cdn0.scrvt.com/39b415fb07de4d9656c7b516d8e2d907/1800000006768945/1ed4126c4f76/Whitepaper‐MR‐only‐RT‐planning‐for‐the‐brain‐and‐pelvis‐with‐synthetic.CT_1800000006768945.pdf. Published 2019. Accessed August 25, 2021.

[68] Lerner M , Medin J , Jamtheim Gustafsson C , Alkner S , Siversson C , Olsson LE . Clinical validation of a commercially available deep learning software for synthetic CT generation for brain. Radiat Oncol. 2021;16:66. 33827619 10.1186/s13014-021-01794-6 PMC8025544

[69] Lei Y , Harms J , Wang T , et al. MRI‐only based synthetic CT generation using dense cycle consistent generative adversarial networks. Med Phys. 2019;46:3565‐3581. 31112304 10.1002/mp.13617 PMC6692192

[70] Peng Y , Chen S , Qin A , et al. Magnetic resonance‐based synthetic computed tomography images generated using generative adversarial networks for nasopharyngeal carcinoma radiotherapy treatment planning. Radiother Oncol. 2020;150:217‐224. 32622781 10.1016/j.radonc.2020.06.049

[71] Emami H , Dong M , Nejad‐Davarani SP , Glide‐Hurst CK . Generating synthetic CTs from magnetic resonance images using generative adversarial networks. Med Phys. 2018;45:3627‐3636. 10.1002/mp.13047 PMC6294710 29901223

[72] Gholamiankhah F , Mostafapour S , Arabi H . Deep learning‐based synthetic CT generation from MR images: comparison of generative adversarial and residual neural networks. Int J Radiat Res. 2022;20:121‐130.

[73] Schmidt MA , Payne GS . Radiotherapy planning using MRI. Phys Med Biol. 2015;60:R323‐R361. 26509844 10.1088/0031-9155/60/22/R323 PMC5137785

[74] Freedman JN , Bainbridge HE , Nill S , et al. Synthetic 4D‐CT of the thorax for treatment plan adaptation on MR‐guided radiotherapy systems. Phys Med Biol. 2019;64:115005. 30844775 10.1088/1361-6560/ab0dbb PMC8208601

[75] Lundervold AS , Lundervold A . An overview of deep learning in medical imaging focusing on MRI. Zeitschrift Fur Medizinische Physik. 2019;29:102‐127. 30553609 10.1016/j.zemedi.2018.11.002

[76] Hunt A , Hansen VN , Oelfke U , Nill S , Hafeez S . Adaptive radiotherapy enabled by MRI guidance. Clin Oncol. 2018;30:711‐719. 10.1016/j.clon.2018.08.001 30201276

[77] Molitoris JK , Diwanji T , Snider JW , et al. Advances in the use of motion management and image guidance in radiation therapy treatment for lung cancer. J Thorac Dis. 2018;10:S2437‐S2450. 30206490 10.21037/jtd.2018.01.155 PMC6123191

[78] Corradini S , Alongi F , Andratschke N , et al. MR‐guidance in clinical reality: current treatment challenges and future perspectives. Radiat Oncol. 2019;14:92. 31167658 10.1186/s13014-019-1308-y PMC6551911

[79] Stemkens B , Tijssen RHN , de Senneville BD , Lagendijk JJW , van den Berg CAT . Image‐driven, model‐based 3D abdominal motion estimation for MR‐guided radiotherapy. Phys Med Biol. 2016;61:5335‐5355. 27362636 10.1088/0031-9155/61/14/5335

[80] Keall PJ , Sawant A , Berbeco RI , et al. AAPM task group 264: the safe clinical implementation of MLC tracking in radiotherapy. Med Phys. 2021;48:e44‐e64. 33260251 10.1002/mp.14625

[81] Borman PTS , Tijssen RHN , Bos C , Moonen CTW , Raaymakers BW , Glitzner M . Characterization of imaging latency for real‐time MRI‐guided radiotherapy. Phys Med Biol. 2018;63:155023. 29995645 10.1088/1361-6560/aad2b7

[82] Glitzner M , Woodhead PL , Borman PTS , Lagendijk JJW , Raaymakers BW . Technical note: MLC‐tracking performance on the Elekta Unity MRI‐linac. Phys Med Biol. 2019;64:15NT02. 10.1088/1361-6560/ab2667 31158831

[83] Fast M , van de Schoot A , van de Lindt T , Carbaat C , van der Heide U , Sonke JJ . Tumor trailing for liver SBRT on the MR‐linac. Int J Radiat Oncol Biol Phys. 2019;103:468‐478. 30243573 10.1016/j.ijrobp.2018.09.011

[84] Dietz B , Yip E , Yun J , Fallone BG , Wachowicz K . Real‐time dynamic MR image reconstruction using compressed sensing and principal component analysis (CS‐PCA): demonstration in lung tumor tracking: demonstration. Med Phys. 2017;44:3978‐3989. 28543069 10.1002/mp.12354

[85] Rudra S , Jiang N , Rosenberg SA , et al. Using adaptive magnetic resonance image‐guided radiation therapy for treatment of inoperable pancreatic cancer. Cancer Med. 2019;8:2123‐2132. 30932367 10.1002/cam4.2100 PMC6536981

[86] Hassanzadeh C , Rudra S , Bommireddy A , et al. Ablative five‐fraction stereotactic body radiation therapy for inoperable pancreatic cancer using online MR‐guided adaptation. Adv Radiat Oncol. 2021;6:100506. 33665480 10.1016/j.adro.2020.06.010 PMC7897757

[87] Green M , van Nest SJ , Soisson E , et al. Three discipline collaborative radiation therapy (3DCRT) special debate: we should treat all cancer patients with hypofractionation. J Appl Clin Med Phys. 2020;21:7‐14. 10.1002/acm2.12954 PMC7324689 32602186

[88] Stemkens B , Paulson ES , Tijssen RHN . Nuts and bolts of 4D‐MRI for radiotherapy. Phys Med Biol. 2018;63:21TR01. 10.1088/1361-6560/aae56d 30272573

[89] Freedman JN , Collins DJ , Gurney‐Champion OJ , et al. Super‐resolution T2‐weighted 4D MRI for image guided radiotherapy. Radiother Oncol. 2018;129:486‐493. 29871813 10.1016/j.radonc.2018.05.015 PMC6294732

[90] Tran EH , Eiben B , Wetscherek A , et al. Evaluation of MRI‐derived surrogate signals to model respiratory motion. Biomed Phys Eng Express. 2020;6:045015. 33194224 10.1088/2057-1976/ab944c PMC7655234

[91] Feng L , Axel L , Chandarana H , Block KT , Sodickson DK , Otazo R . XD‐GRASP: Golden‐angle radial MRI with reconstruction of extra motion‐state dimensions using compressed sensing. Magn Reson Med. 2016;75:775‐788. 25809847 10.1002/mrm.25665 PMC4583338

[92] Johansson A , Balter JM , Cao Y . Gastrointestinal 4D MRI with respiratory motion correction. Med Phys. 2021;48:2521‐2527. 33595909 10.1002/mp.14786 PMC8172093

[93] Kovacs B , Mayinger M , Tanadini‐Lang S , et al. First two MRI guided stereotactic body radiation therapy of recurrent sustained ventricular tachycardia. Eur Heart J. 2020;41:ehaa946.0758.

[94] Keall PJ , Mageras GS , Balter JM , et al. The management of respiratory motion in radiation oncology report of AAPM task group 76a. Med Phys. 2006;33:3874‐3900. 17089851 10.1118/1.2349696

[95] Otazo R , Lambin P , Pignol J‐P , et al. MRI‐guided radiation therapy: An emerging paradigm in adaptive radiation oncology. Radiology. 2021;298:248‐260. 33350894 10.1148/radiol.2020202747 PMC7924409

[96] McClelland JR , Hawkes DJ , Schaeffter T , King AP . Respiratory motion models: a review. Med Image Anal. 2013;17:19‐42. 23123330 10.1016/j.media.2012.09.005

[97] Zhang Y , Kashani R , Cao Y , et al. A hierarchical model of abdominal configuration changes extracted from golden angle radial magnetic resonance imaging. Phys Med Biol. 2021;66:045018. 33361579 10.1088/1361-6560/abd66e PMC7993537

[98] Mickevicius NJ , Paulson ES . On the use of low‐dimensional temporal subspace constraints to reduce reconstruction time and improve image quality of accelerated 4D‐MRI. Radiother Oncol. 2021;158:215‐223. 33412207 10.1016/j.radonc.2020.12.032

[99] Freedman JN , Gurney‐Champion OJ , Nill S , et al. Rapid 4D‐MRI reconstruction using a deep radial convolutional neural network: Dracula. Radiother Oncol. 2021;159:209‐217. 33812914 10.1016/j.radonc.2021.03.034 PMC8216429

[100] Waddington DEJ , Hindley N , Koonjoo N , et al. On real‐time image reconstruction with neural networks for MRI‐guided radiotherapy. arXiv preprint arXiv:2202.05267 2022.

[101] Terpstra ML , Maspero M , D'Agata F , et al. Deep learning‐based image reconstruction and motion estimation from undersampled radial k‐space for real‐time MRI‐guided radiotherapy. Phys Med Biol. 2020;65:155015. 32408295 10.1088/1361-6560/ab9358

[102] Hyun CM , Kim HP , Lee SM , Lee S , Seo JK . Deep learning for undersampled MRI reconstruction. Phys Med Biol. 2018;63:135007. 29787383 10.1088/1361-6560/aac71a

[103] van Houdt PJ , Yang Y , van der Heide UA . Quantitative magnetic resonance imaging for biological image‐guided adaptive radiotherapy. Front Oncol. 2021;10:615643. 10.3389/fonc.2020.615643 PMC7878523 33585242

[104] Thompson SM , Gorny KR , Koepsel EMK , et al. Body interventional MRI for diagnostic and interventional radiologists: current practice and future prospects. Radiographics. 2021;41:1785‐1801. 34597216 10.1148/rg.2021210040

[105] Hoffmann A , Oborn B , Moteabbed M , et al. MR‐guided proton therapy: a review and a preview. Radiat Oncol. 2020;15:1‐13. 10.1186/s13014-020-01571-x PMC7260752 32471500

[106] Gurney‐Champion OJ , Mahmood F , van Schie M , et al. Quantitative imaging for radiotherapy purposes. Radiother Oncol. 2020;146:66‐75. 32114268 10.1016/j.radonc.2020.01.026 PMC7294225

[107] Das IJ , McGee KP , Tyagi N , Wang H . Role and future of MRI in radiation oncology. Br J Radiol. 2019;92:20180505. 30383454 10.1259/bjr.20180505 PMC6404845

[108] Arai TJ , Yang DM , Campbell JW , et al. Oxygen‐sensitive MRI: A predictive imaging biomarker for tumor radiation response? Int J Radiat Oncol Biol Phys. 2021;110:1519‐1529. 33775857 10.1016/j.ijrobp.2021.03.039 PMC8286313

[109] Datta A , Aznar MC , Dubec M , Parker GJM , O'Connor JPB . Delivering functional imaging on the MRI‐linac: current challenges and potential solutions. Clin Oncol. 2018;30:702‐710. 10.1016/j.clon.2018.08.005 30224203

[110] Tatum JL . Hypoxia: importance in tumor biology, noninvasive measurement by imaging, and value of its measurement in the management of cancer therapy. Int J Radiat Biol. 2006;82:699‐757. 17118889 10.1080/09553000601002324

[111] Paech D , Nagel AM , Schultheiss MN , et al. Quantitative dynamic oxygen 17 MRI at 7.0 T for the cerebral oxygen metabolism in glioma. Radiology. 2020;295:181‐189. 32068505 10.1148/radiol.2020191711

[112] Scott JG , Sedor G , Ellsworth P , et al. Pan‐cancer prediction of radiotherapy benefit using genomic‐adjusted radiation dose (GARD): a cohort‐based pooled analysis. Lancet Oncol. 2021;22:1221‐1229. 34363761 10.1016/S1470-2045(21)00347-8 PMC12818176

[113] Tomaszewski MR , Latifi K , Boyer E , et al. Delta radiomics analysis of magnetic resonance guided radiotherapy imaging data can enable treatment response prediction in pancreatic cancer. Radiat Oncol. 2021;16:237. 34911546 10.1186/s13014-021-01957-5 PMC8672552

[114] Tahir BA , Hughes PJC , Robinson SD , et al. Spatial comparison of CT‐based surrogates of lung ventilation with hyperpolarized helium‐3 and xenon‐129 gas MRI in patients undergoing radiation therapy. Int J Radiat Oncol Biol Phys. 2018;102:1276‐1286. 30355463 10.1016/j.ijrobp.2018.04.077

[115] Ireland RH , Tahir BA , Wild JM , Lee CE , Hatton MQ . Functional image‐guided radiotherapy planning for normal lung avoidance. Clin Oncol. 2016;28:695‐707. 10.1016/j.clon.2016.08.005 27637724

[116] Ireland RH , Din OS , Swinscoe JA , et al. Detection of radiation‐induced lung injury in non‐small cell lung cancer patients using hyperpolarized helium‐3 magnetic resonance imaging. Radiother Oncol. 2010;97:244‐248. 20724011 10.1016/j.radonc.2010.07.013

[117] Ireland RH , Bragg CM , McJury M , et al. Feasibility of image registration and intensity‐modulated radiotherapy planning with hyperpolarized helium‐3 magnetic resonance imaging for non–small‐cell lung cancer. Int J Radiat Oncol Biol Phys. 2007;68:273‐281. 17448880 10.1016/j.ijrobp.2006.12.068 PMC2713782

[118] O'Connor JPB , Aboagye EO , Adams JE , et al. Imaging biomarker roadmap for cancer studies. Nat Rev Clin Oncol. 2017;14:169‐186. 27725679 10.1038/nrclinonc.2016.162 PMC5378302

[119] van Houdt PJ , Saeed H , Thorwarth D , et al. Integration of quantitative imaging biomarkers in clinical trials for MR‐guided radiotherapy: conceptual guidance for multicentre studies from the MR‐Linac consortium imaging biomarker working group. Eur J Cancer. 2021;153:64‐71. 34144436 10.1016/j.ejca.2021.04.041 PMC8340311

[120] Verkooijen HM , Kerkmeijer LGW , Fuller CD , et al. R‐IDEAL: a framework for systematic clinical evaluation of technical innovations in radiation oncology. Front Oncol. 2017;7:59. 28421162 10.3389/fonc.2017.00059 PMC5378068

[121] Mierzwa ML , Gharzai LA , Li P , et al. Early MRI blood volume changes in constrictor muscles correlate with postradiation dysphagia. Int J Radiat Oncol Biol Phys. 2021;110:566‐573. 33346093 10.1016/j.ijrobp.2020.12.018 PMC12273593

[122] Keenan KE , Biller JR , Delfino JG , et al. Recommendations towards standards for quantitative MRI (qMRI) and outstanding needs. J Magn Reson Imaging. 2019;49:e26‐e39. 30680836 10.1002/jmri.26598 PMC6663309

[123] Yankeelov T . The quantitative imaging network: a decade of achievement. Tomography. 2019;5:A8. 30854462 10.18383/j.tom.2019.00999 PMC6403024

[124] Shukla‐Dave A , Obuchowski NA , Chenevert TL , et al. Quantitative imaging biomarkers alliance (QIBA) recommendations for improved precision of DWI and DCE‐MRI derived biomarkers in multicenter oncology trials. J Magn Reson Imaging. 2019;49:e101‐e121. 30451345 10.1002/jmri.26518 PMC6526078

[125] Klüter S . Technical design and concept of a 0.35 T MR‐Linac. Clin Transl Radiat Oncol. 2019;18:98‐101. 31341983 10.1016/j.ctro.2019.04.007 PMC6630153

[126] Lagendijk JJW , van Vulpen M , Raaymakers BW . The development of the MRI linac system for online MRI‐guided radiotherapy: a clinical update. J Intern Med. 2016;280:203‐208. 27197553 10.1111/joim.12516

[127] Wiedenmann N , Grosu A‐L , Büchert M , et al. The utility of multiparametric MRI to characterize hypoxic tumor subvolumes in comparison to FMISO PET/CT. Consequences for diagnosis and chemoradiation treatment planning in head and neck cancer. Radiother Oncol. 2020;150:128‐135. 32544609 10.1016/j.radonc.2020.06.013

[128] Hompland T , kon Hole KH , Ragnum HB , et al. Combined MR imaging of oxygen consumption and supply reveals tumor hypoxia and aggressiveness in prostate cancer patients. Cancer Res. 2018;78:4774‐4785. 29945958 10.1158/0008-5472.CAN-17-3806

[129] Hillestad T , Hompland T , Fjeldbo CS , et al. MRI distinguishes tumor hypoxia levels of different prognostic and biological significance in cervical cancer. Cancer Res. 2020;80:3993‐4003. 32606004 10.1158/0008-5472.CAN-20-0950

[130] Harris RJ , Cloughesy TF , Liau LM , et al. PH‐weighted molecular imaging of gliomas using amine chemical exchange saturation transfer MRI. Neuro Oncol. 2015;17:1514‐1524. 26113557 10.1093/neuonc/nov106 PMC4648305

[131] Saxena K , Jolly MK . Acute vs. chronic vs. cyclic hypoxia: their differential dynamics, molecular mechanisms, and effects on tumor progression. Biomolecules. 2019;9:339. 31382593 10.3390/biom9080339 PMC6722594

[132] Panek R , Welsh L , Baker LCJ , et al. Noninvasive imaging of cycling hypoxia in head and neck cancer using intrinsic susceptibility MRI. Clin Cancer Res. 2017;23:4233‐4241. 28314789 10.1158/1078-0432.CCR-16-1209 PMC5516915

[133] Muthana M , Kennerley AJ , Hughes R , et al. Directing cell therapy to anatomic target sites in vivo with magnetic resonance targeting. Nat Commun. 2015;6:8009. 26284300 10.1038/ncomms9009 PMC4568295

[134] Graham K , Unger E . Overcoming tumor hypoxia as a barrier to radiotherapy, chemotherapy and immunotherapy in cancer treatment. Int J Nanomedicine. 2018;13:6049. 30323592 10.2147/IJN.S140462 PMC6177375

[135] Maraghechi B , Gach HM , Setianegara J , Yang D , Li HH . Dose uncertainty and resolution of polymer gel dosimetry using an MRI guided radiation therapy system's onboard 0.35 T scanner. Phys Med. 2020;73:8‐12. 32279048 10.1016/j.ejmp.2020.04.004 PMC11449075

[136] Ma D , Gulani V , Seiberlich N , et al. Magnetic resonance fingerprinting. Nature. 2013;495:187‐192. 23486058 10.1038/nature11971 PMC3602925

[137] Fessler J . Model‐based image reconstruction for MRI. IEEE Signal Process Mag. 2010;27:81‐89. 21135916 10.1109/MSP.2010.936726 PMC2996730

[138] van der Heide O , Sbrizzi A , van den Berg CAT . Accelerated MR‐STAT reconstructions using sparse hessian approximations. IEEE Trans Med Imaging. 2020;39:3737‐3748. 32746119 10.1109/TMI.2020.3003893

[139] Liu F , Feng L , Kijowski R . MANTIS: model‐augmented neural neTwork with incoherent k‐space sampling for efficient MR parameter mapping. Magn Reson Med. 2019;82:174‐188. 30860285 10.1002/mrm.27707 PMC7144418

[140] Lønning K , Putzky P , Sonke JJ , Reneman L , Caan MWA , Welling M . Recurrent inference machines for reconstructing heterogeneous MRI data. Med Image Anal. 2019;53:64‐78. 30703579 10.1016/j.media.2019.01.005

[141] Barbieri S , Gurney‐Champion OJ , Klaassen R , Thoeny HC . Deep learning how to fit an intravoxel incoherent motion model to diffusion‐weighted MRI. Magn Reson Med. 2020;83:312‐321. 31389081 10.1002/mrm.27910

[142] Ulas C , Tetteh G , Thrippleton MJ , et al. Direct estimation of pharmacokinetic parameters from DCE‐MRI using deep CNN with forward physical model loss. Lecture Notes in Computer Science (including subseries Lecture Notes in Artificial Intelligence and Lecture Notes in Bioinformatics) 2018;11070 LNCS:39–47.

[143] Kaandorp MPT , Barbieri S , Klaassen R , et al. Improved unsupervised physics‐informed deep learning for intravoxel incoherent motion modeling and evaluation in pancreatic cancer patients. Magn Reson Med. 2021;86:2250‐2265. 34105184 10.1002/mrm.28852 PMC8362093

[144] Geethanath S , Baek H‐M , Ganji SK , et al. Compressive sensing could accelerate 1H MR metabolic imaging in the clinic. Radiology. 2012;262:985‐994. 22357898 10.1148/radiol.11111098 PMC3285227

[145] Speight R , Dubec M , Eccles CL , et al. IPEM topical report: guidance on the use of MRI for external beam radiotherapy treatment planning. Phys Med Biol. 2021;66:055025. 10.1088/1361-6560/abdc30 33450742

[146] Shan S , Liney GP , Tang F , et al. Geometric distortion characterization and correction for the 1.0 T Australian MRI‐linac system using an inverse electromagnetic method. Med Phys. 2020;47:1126‐1138. 31856301 10.1002/mp.13979

[147] Walker A , Liney G , Metcalfe P , Holloway L . MRI distortion: considerations for MRI based radiotherapy treatment planning. Australas Phys Eng Sci Med. 2014;37:103‐113. 24519001 10.1007/s13246-014-0252-2

[148] Crijns SPM , Raaymakers BW , Lagendijk JJW . Real‐time correction of magnetic field inhomogeneity‐induced image distortions for MRI‐guided conventional and proton radiotherapy. Phys Med Biol. 2011;56:289‐297. 21149949 10.1088/0031-9155/56/1/017

[149] Medicines and Healthcare Products Regulatory Agency (MHRA), Safety Guidelines for Magnetic Resonance Imaging Equipment in Clinical Use 2021.

[150] O'Reilly T , Webb A . Deconstructing and reconstructing MRI hardware. J Magn Reson. 2019;306:134‐138. 31311711 10.1016/j.jmr.2019.07.014

[151] Campbell‐Washburn AE , Xue H , Lederman RJ , Faranesh AZ , Hansen MS . Real‐time distortion correction of spiral and echo planar images using the gradient system impulse response function. Magn Reson Med. 2016;75:2278‐2285. 26114951 10.1002/mrm.25788 PMC4691439

[152] Koonjoo N , Zhu B , Bagnall GC , Bhutto D , Rosen MS . Boosting the signal‐to‐noise of low‐field MRI with deep learning image reconstruction. Sci Rep. 2021;11:8248. 33859218 10.1038/s41598-021-87482-7 PMC8050246

[153] Mutic S , Dempsey JF . The ViewRay system: magnetic resonance‐guided and controlled radiotherapy. Semin Radiat Oncol. 2014;24:196‐199. 24931092 10.1016/j.semradonc.2014.02.008

[154] Raaymakers BW , De Boer JCJ , Knox C , et al. Integrated megavoltage portal imaging with a 1.5 T MRI linac. Phys Med Biol. 2011;56:N207‐N214. 10.1088/0031-9155/56/19/N01 21934191

[155] Tijssen RHN , Philippens MEP , Paulson ES , et al. MRI commissioning of 1.5T MR‐linac systems – a multi‐institutional study. Radiother Oncol. 2019;132:114‐120. 30825959 10.1016/j.radonc.2018.12.011

[156] Fallone BG , Murray B , Rathee S , et al. First MR images obtained during megavoltage photon irradiation from a prototype integrated linac‐MR system. Med Phys. 2009;36:2084‐2088. 19610297 10.1118/1.3125662

[157] Keall PJ , Barton M , Crozier S . The Australian magnetic resonance imaging‐linac program. Semin Radiat Oncol. 2014;24:203‐206. 24931094 10.1016/j.semradonc.2014.02.015

[158] Dayarian I , Chan TCY , Jaffray D , Stanescu T . A mixed‐integer optimization approach for homogeneous magnet design. Technology. 2018;06:49‐58.

[159] Moritz M , Redlich T , Günyar S , Winter L , Wulfsberg JP . On the economic value of open source hardware – case study of an open source magnetic resonance imaging scanner. J Open Hardware. 2019;3:2.

[160] Hoogcarspel SJ , Crijns SPM , Lagendijk JJW , Van Vulpen M , Raaymakers BW . The feasibility of using a conventional flexible RF coil for an online MR‐guided radiotherapy treatment. Phys Med Biol. 2013;58:1925‐1932. 23442765 10.1088/0031-9155/58/6/1925

[161] Ghila A , Fallone BG , Rathee S . Influence of standard RF coil materials on surface and buildup dose from a 6 MV photon beam in magnetic field. Med Phys. 2016;43:5808‐5816. 27806597 10.1118/1.4963803

[162] Zijlema SE , Tijssen RHN , Malkov VN , et al. Design and feasibility of a flexible, on‐body, high impedance coil receive array for a 1.5 T MR‐linac. Phys Med Biol. 2019;64:185004. 10.1088/1361-6560/ab37a8 31370043

[163] Zijlema SE , Tijssen RHN , van Dijk L , et al. Improving the imaging performance of the 1.5 T MR‐linac using a flexible, 32‐channel, on‐body receive array. Phys Med Biol. 2020;65:215008. 10.1088/1361-6560/aba87a 32698168

[164] Zhang B , Brown R , Cloos M , Lattanzi R , Sodickson D , Wiggins G . Size‐adaptable “Trellis” structure for tailored MRI coil arrays. Magn Reson Med. 2019;81:3406‐3415. 30575119 10.1002/mrm.27637 PMC6484426

[165] Corea JR , Flynn AM , Lechêne B , et al. Screen‐printed flexible MRI receive coils. Nat Commun. 2016;7:1‐7. 10.1038/ncomms10839 PMC5553354 26961073

[166] Collick BD , Behzadnezhad B , Hurley SA , et al. Rapid development of application‐specific flexible MRI receive coils. Phys Med Biol. 2020;65:19NT01. 10.1088/1361-6560/abaffb PMC8064628 32975219

[167] Alsadah A , van Merode T , Alshammari R , Kleijnen J . A systematic literature review looking for the definition of treatment burden. Heliyon. 2020;6:e03641. 10.1016/j.heliyon.2020.e03641 PMC7150517 32300666

[168] El‐Turk N , Chou MSH , Ting NCH , et al. Treatment burden experienced by patients with lung cancer. PLOS One. 2021;16:e0245492. 33481895 10.1371/journal.pone.0245492 PMC7822249

[169] Perrier L , Morelle M , Pommier P , et al. Cost of prostate image‐guided radiation therapy: results of a randomized trial. Radiother Oncol. 2013;106:50‐58. 23333022 10.1016/j.radonc.2012.11.011

[170] Tetar SU , Bruynzeel AME , Lagerwaard FJ , Slotman BJ , Bohoudi O , Palacios MA . Clinical implementation of magnetic resonance imaging guided adaptive radiotherapy for localized prostate cancer. Phys Imaging Radiat Oncol. 2019;9:69‐76. 33458428 10.1016/j.phro.2019.02.002 PMC7807673

[171] Stanescu T , Shessel A , Carpino‐Rocca C , et al. MRI‐guided online adaptive stereotactic body radiation therapy of liver and pancreas tumors on an MR‐linac system. Cancers. 2022;14:716. 35158984 10.3390/cancers14030716 PMC8833602

[172] Sayan M , Serbez I , Teymur B , et al. Patient‐reported tolerance of magnetic resonance‐guided radiation therapy. Front Oncol. 2020;10:1782. 10.3389/fonc.2020.01782 PMC7537416 33072560

[173] Barnes H , Alexander S , Bower L , et al. Development and results of a patient‐reported treatment experience questionnaire on a 1.5 T MR‐Linac. Clin Transl Radiat Oncol. 2021;30:31‐37. 34307911 10.1016/j.ctro.2021.06.003 PMC8283148

[174] Meléndez JC , McCrank E . Anxiety‐related reactions associated with magnetic resonance imaging examinations. JAMA. 1993;270:745. 8336378 10.1001/jama.1993.03510060091039

[175] Liu PZY , Dong B , Nguyen DT , et al. First experimental investigation of simultaneously tracking two independently moving targets on an MRI‐linac using real‐time MRI and MLC tracking. Med Phys. 2020;47:6440‐6449. 33058211 10.1002/mp.14536

[176] Zilli T , Scorsetti M , Zwahlen D , et al. ONE SHOT ‐ single SHOT radiotherapy for localized prostate cancer: study protocol of a single arm, multicenter phase I/II trial. Radiat Oncol. 2018;13:166. 30180867 10.1186/s13014-018-1112-0 PMC6123974

[177] Cuccia F , Corradini S , Mazzola R , et al. MR‐guided hypofractionated radiotherapy: current emerging data and promising perspectives for localized prostate cancer. Cancers. 2021;13:1791. 33918650 10.3390/cancers13081791 PMC8070332

[178] Hannan R , Tumati V , Xie X‐J , et al. Stereotactic body radiation therapy for low and intermediate risk prostate cancer—results from a multi‐institutional clinical trial. Eur J Cancer. 2016;59:142‐151. 27035363 10.1016/j.ejca.2016.02.014

[179] Videtic GMM , Hu C , Singh AK , et al. A randomized phase 2 study comparing 2 stereotactic body radiation therapy schedules for medically inoperable patients with stage I peripheral non‐small cell lung cancer: NRG oncology RTOG 0915 (NCCTG N0927). Int J Radiat Oncol Biol Phys. 2015;93:757‐764. 26530743 10.1016/j.ijrobp.2015.07.2260 PMC4744654

[180] Rahimi A , Thomas K , Spangler A , et al. Preliminary results of a phase 1 dose‐escalation trial for early‐stage breast cancer using 5‐fraction stereotactic body radiation therapy for partial‐breast irradiation. Int J Radiat Oncol Biol Phys. 2017;98:196‐205.e2. 28586960 10.1016/j.ijrobp.2017.01.020

[181] Lievens Y , Defourny N , Coffey M , et al. Radiotherapy staffing in the European countries: final results from the ESTRO‐HERO survey. Radiother Oncol. 2014;112:178‐186. 25300718 10.1016/j.radonc.2014.08.034

[182] Bellometti S , Nube G , Alongi F , et al. Radiotherapy activities and technological equipment in Veneto, Italy: a report from the rete Radioterapica Veneta. Radiol Med. 2021;126:623‐629. 33242206 10.1007/s11547-020-01308-6

[183] Korreman S , Eriksen JG , Grau C . The changing role of radiation oncology professionals in a world of AI – just jobs lost – or a solution to the under‐provision of radiotherapy? Clin Transl Radiat Oncol. 2021;26:104‐107. 33364449 10.1016/j.ctro.2020.04.012 PMC7752957

[184] McIntosh C , Conroy L , Tjong MC , et al. Clinical integration of machine learning for curative‐intent radiation treatment of patients with prostate cancer. Nat Med. 2021;27:999‐1005. 34083812 10.1038/s41591-021-01359-w

[185] Willmann J , Poortmans P , Monti AF , et al. Development of staffing, workload and infrastructure in member departments of the European Organisation for research and treatment of cancer (EORTC) radiation oncology group. Radiother Oncol. 2021;155:226‐231. 33217496 10.1016/j.radonc.2020.11.009

